# Absence of pesticide avoidance during chronic colony-level exposure modifies locomotor activity in bumble bees

**DOI:** 10.1007/s10646-026-03045-4

**Published:** 2026-02-21

**Authors:** Lívia Maria Negrini Ferreira, Gaetana Mazzeo, Maria Augusta Pereira Lima

**Affiliations:** 1 Departamento de Entomologia, Universidade Federal de Viçosa, Viçosa, MG Brazil; 2 Dipartimento di Agricoltura, Alimentazione e Ambiente, Università degli Studi di Catania, Catania, CT Italy; 3 Departamento de Biologia Animal, Universidade Federal de Viçosa, Viçosa, MG Brazil

**Keywords:** *Bombus terrestris*, Food deterrence, Foraging behavior, Pesticide contaminated food, Sublethal effects

## Abstract

Exposure to pesticides partly depends on the foraging behavior of bees, which may exhibit indifference, deterrence, or attraction to contaminated food. In the present study, we conducted laboratory experiments to test the foraging avoidance of *Bombus terrestris* for honey syrup contaminated with field-realistic concentrations of the neonicotinoid acetamiprid (ACE), herbicide glyphosate (GLY), and fungicide metalaxyl-M (MET). Tests were also conducted with the recommended field concentration of a biopesticide, the sweet orange essential oil (EOE). Bees’ behavior, and lethal and sublethal effects of the pesticides on bumble bees were assessed at the individual (isolated foragers) and colony (colony exposure) levels. Bees did not display any avoidance for contaminated or uncontaminated food at the individual or colony levels in the ACE, GLY, and MET treatments. However, the EOE treatment reduced the consumption of honey syrup in relation to non-treated bees, resulting in lower survival of individual bees. At the individual level, no behavioral differences were observed between non-treated and treated bees. At the colony level, however, pesticide treatments modified bees’ walking behavior. In general, pesticides increased resting time and meandering, while reducing speed, movement, distance walked, and time spent moving fast. Our results demonstrate that bumble bees did not reduce food consumption of syrup contaminated with realistic concentrations of different common pesticides, resulting in detrimental effects on their behavior. Furthermore, bumble bees treated with the biopesticide field concentration reduced food consumption both at individual and colony levels. This treatment caused negative behavioral changes on bees and decreased the survival of isolated individuals.

**Supplementary Information:**

The online version contains supplementary material available at 10.1007/s10646-026-03045-4.

## Introduction

While foraging on food cultivars and flowering weeds near or within crops, bees can be exposed to many pesticides, and different species may react differently to the environmental factors introduced by human activity (Rodrigues et al. 2018; Ward et al. 2022). Exposure to pesticides is partly influenced by foraging behavior of bees, which may exhibit indifference, deterrence, or attraction to contaminated food (Arce et al. 2018; Ferreira et al. 2024). The inability of bees to avoid plants treated with pesticides leads to the collection of contaminated nectar and pollen, which are then taken to the colony, exposing not only the foragers, but the entire colony (Motta and Moran 2023; Zioga et al. 2023).

The collection of contaminated resources by bees is confirmed by the presence of pesticide residues in food stored in the colonies (Botías et al. 2017; Kiljanek et al. 2017; Thompson et al. 2019; Main et al. 2020; Rondeau and Raine 2022; Zioga et al. 2023; Nicholson et al. 2024). Residue levels vary among bee species. In some cases, bumble bees (Apidae: Bombini) show greater pesticide exposure than honey bees (Apidae: Apini), including detectable residues in queens (Main et al. 2020; Zioga et al. 2023). Moreover, bumble bees do not seem to avoid food contaminated with pesticides (Muth et al. 2020; Kuivila et al. 2021; Thompson et al. 2022; Motta and Moran 2023; Nicholson et al. 2024), and in some cases, they may even be attracted to resources contaminated with these products (Kessler et al. 2015; Arce et al. 2018; Rondeau 2024).

To date, the preference of bumble bees for food contaminated with realistic concentrations of pesticides has only been studied in laboratory with neonicotinoids (Kessler et al. 2015; Arce et al. 2018; Muth et al. 2020; Ferreira et al. 2024). However, in the field, these bees are exposed to several other groups of pesticides (Botías et al. 2017; Kiljanek et al. 2017; Main et al. 2020; Nicholson et al. 2024), which can cause sublethal effects on individual bees and interfere with colony development (Barbosa et al. 2015; Seide et al. 2018; Cappa et al. 2022; Rondeau and Raine 2022; Tosi et al. 2022; Souza et al. 2023; Strang et al. 2024). Among the sublethal effects observed in bumble bees are behavioral modifications such as foraging impairments, which can alter pollinator-plant interactions and colony maintenance due to exposure to neonicotinoids (Stanley and Raine 2016), biopesticides (Barbosa et al. 2015), herbicides (Nouvian et al. 2023), and fungicides (Tamburini et al. 2021).

In social bees, foraging at the individual level does not necessarily reflect foraging at the colony level, due to task allocation and social interactions (Hendriksma et al. 2019). Therefore, it is important to understand colony and individual foraging decisions to evaluate the probability of pesticide exposure in bumble bee populations (Gradish et al. 2019; Main et al. 2020). For instance, although neonicotinoid-contaminated bumble bee foragers increased their visits to flowers, the exposed colonies had overall lower visitation rates (Stanley et al. 2015). In addition, even though behavioral changes in response to pesticides might not be observed at the individual level, they can be observed at the colony level (Weidenmüller et al. 2022). Therefore, assessment of pesticide effects on bumble bees at the colony and individual levels is important to understand their impacts on bee populations and pollinator-plant interactions (Demirozer et al. 2022).

In this study, under controlled laboratory conditions, we investigated the foraging avoidance of *Bombus terrestris* Linnaeus, 1758 (Apidae: Bombini) when exposed to food contaminated with different types of pesticides. Bee avoidance was assessed at the colony and individual levels. Foragers of this bumble bee species were allowed to choose between pure honey syrup or food sources containing field-realistic doses of acetamiprid (neonicotinoid insecticide), sweet orange essential oil (biopesticide), glyphosate (herbicide), or metalaxyl-M (fungicide). We aimed to answer the following questions: (1) Do bumble bees exhibit avoidance for any of the offered pesticide-contaminated food sources over uncontaminated food? (2) Do the foraging behavior varies between individuals and colonies of bumble bees submitted to food choice tests with pesticides? (3) Does the choice to ingest contaminated or uncontaminated food have lethal or sublethal effects on individuals and colonies of bumble bees?

## Materials and methods

### Bees and pesticides

Colonies of *B. terrestris* were purchased from Koppert Biological Systems (Netherlands) from September 2023 to April 2024. The colonies belonged to the Natupol Excel line, which contained one large colony of bumble bees, including a queen, workers, brood, and sugar water. Sugar water was removed for the subsequent experiments so that bees could collect liquid food only from the feeders provided. The colonies were kept inside a cage (BugDorm-4E4590DH Specimen Handling Cage; Dimensions: W93.0 × D47.5 × H47.5 cm; Main Material: Woven Nylon Netting), and the bees were free to leave the nest and collect honey syrup (1:1 v/v honey and distilled water solution) offered in bee feeders inside the cage. Commercial honey bee pollen was provided once a week and deposited inside the colonies. The pollen used was not analyzed for pesticide residues prior to the experiment; however, it was homogenized and consistently used across all treatments, including the negative control. The experimental period was limited to four days, as bees can exhibit measurable food preference or avoidance responses within a short time frame.

We selected four active ingredients used in cultivars visited by bumble bees: the neonicotinoid insecticide acetamiprid (apple blossoms, berries, greenhouse tomato, pepper) the biopesticide sweet orange essential oil (strawberry, blueberry, greenhouse tomato and pepper), the herbicide glyphosate (orchard blossoms), and the fungicide metalaxyl-M (greenhouse tomato and pepper, sunflower). Acetamiprid, glyphosate, and metalaxyl-M are systemic pesticides, and their residues have been found in plants visited by bees, as well as in the body of these insects (Kiljanek et al. 2017; Thompson et al. 2019; El Agrebi et al. 2020; Zioga et al. 2020, 2023; Rondeau and Raine 2022). Sweet orange essential oil can be deposited in flowers if the plants are treated during the flowering period. The use of these active ingredients is widespread in Europe (Shattuck et al. 2023; European Commission 2025), where *Bombus terrestris* is a native species.

For acetamiprid treatments, we used the commercial formulation Epik^®^ SL (Sipcam, a.i.: 4.67% = 50 g/l). Residues of acetamiprid found in nectar can range from 0.002 to 0.01 µg/ml (Pohorecka et al. 2012; Wen et al. 2021; Azpiazu et al. 2023). Based on this, we used the field-realistic dose of 0.01 µg/ml of acetamiprid in our experiments.

For the metalaxyl-M treatments, we used the commercial formulation Ridomil Gold SL (Syngenta, a.i.: 43.88% = 465 g/l). Residues of metalaxyl-M found in nectar can range from 0.002 to 0.15 µg/ml (Pohorecka et al. 2012; Gong et al. 2020; Wen et al. 2021). Therefore, we used a realistic field dose of 0.05 µg/ml of metalaxyl-M.

For glyphosate treatments, we used the commercial formulation Taifun^®^ MK CL (Adama, Italy, a.i.: 30.8% = 360 g/l). Residues of glyphosate found in nectar can range from 0.1 to 31 µg/ml (Thompson et al. 2014; Zioga et al. 2022, 2023). Based on that, we used the field-realistic dose of 30 µg/ml of glyphosate in our experiments.

For sweet orange essential oil treatments, we used the commercial formulation Prev-Am^®^ Plus (Oroagri International Ltd, Italy, a.i.: 5.88% = 60 g/l). As literature on residues of sweet orange essential oil in nectar was not available, field-realistic dose of 476 µg/ml of sweet orange essential oil was used for further experiments. This is the field dose recommended for tomato crops, simulating biopesticide deposition on flower after biopesticide spraying. Our experimental design aimed to represent a worst-case exposure scenario, in which pollinators encounter pesticide immediately after application. This approach was adopted because some product labels do not explicitly discourage the use of this biopesticide during the flowering period (Rovensa Next 2023; Ascenza 2026; MAPA 2026). Moreover, biopesticides are often perceived as harmless to non-target insects and are therefore assumed to be safe for use at any time, including during flowering (Challa et al. 2019).

### Pesticide avoidance tests

#### Colony-level avoidance tests

The bioassays were performed between September 2023 and May 2024. Each bumble bee colony was isolated inside a translucent cage and had access to two feeders (Qicfrk^®^, 20.6 × 12.9 × 8 cm, 151.1 g, model H0046), one of them had uncontaminated honey syrup and the other had one of the following treatments: (CTRL) - uncontaminated honey syrup (control); (ACE) - honey syrup contaminated with 0.01 µg/ml of acetamiprid; (EOE) - honey syrup contaminated with 476 µg/ml of sweet orange essential oil; (GLY) - honey syrup contaminated with 30 µg/ml of glyphosate; or (MET) - honey syrup contaminated with 0.05 µg/ml of metalaxyl-M. Each colony was a replicate, and each treatment had three replicates, leading to 15 bumble bee colonies used in the experiment.

Each colony received access to two feeders for four consecutive days: one containing uncontaminated honey syrup, and the other with honey syrup contaminated with one of the four pesticide treatments. The feeders were randomly repositioned by drawing lots and cleaned with alcohol every 24 h to avoid positional bias (Arce et al. 2018; Ferreira et al. 2024). The avoidance of bumble bee colonies for contaminated or uncontaminated food was estimated by weighing the feeders before and after each day of exposure to measure food intake. Food evaporation was not measured; however, all individuals and colonies from each treatment group were kept at the same conditions. Laboratory temperature was monitored using a temperature and relative humidity (RH) data logger.

#### Individual-level avoidance tests

Individual-level experiments were performed using the same colonies used for the colony-level experiments, and both experiments were performed simultaneously. Each colony assigned to one of the five treatments (CTRL, ACE, EOE, GLY, or MET) was considered a replicate. From each colony, five workers were collected and assigned to the same pesticide treatment group as the colony of origin, totaling 15 bees per treatment. The individuals were cold-anesthetized by placement in a − 20 °C freezer until immobilization was achieved and weighed, and only those that were not very small (approx. < 0.13 g), or very large (> 0.36 g) were included in the individual-level avoidance test (Sgolastra et al. 2017).

The selected workers were transferred to individual 650 ml plastic cages (W15.0 × D15.0 × H7.0 cm) with a perforated lid and mesh to allow ventilation. Two 3 ml syringes were inserted in the cages horizontally, one containing uncontaminated honey syrup and one containing contaminated honey syrup according to the following treatment conditions: (CTRL) uncontaminated honey syrup (control); (ACE) 0.01 µg/ml of acetamiprid; (EOE) 476 µg/ml of sweet orange essential oil; (GLY) 30 µg/ml of glyphosate; or (MET) 0.05 µg/ml of metalaxyl-M.

The bumble bees had access to honey syrups for four days. The syringes were replaced and repositioned each day. The syringes were weighed before their inclusion into the cages and after daily exposure to calculate individual food consumption. The bumble bees were maintained in a dark room with 22 ± 2 °C and 65% RH.

### Assessment of lethal and sublethal effects

At the colony level, lethal effects were evaluated by counting the number of dead bees outside the nest after the four days of avoidance test. Colonies were weighed before and after the avoidance test to assess sublethal effects on colony weight gain. At the individual level, bee survival was assessed every 24 h for four days to evaluate lethal effects.

After four days of the avoidance test, the bees that survived the individual-level test and five bees collected from each colony that underwent the colony-level test were filmed for behavioral analysis using the Ethoflow^®^ software (Bernardes et al. 2021). The behavioral variables measured were as follows: distance walked (cm), mean walking speed (cm/s), meandering (degrees, the angle average that the individual turned during the video), resting time (s), mean movement time (proportion of time that the insects remained in intermediate activity: distance walked > 0.07 and ≤ 0.4 cm/frame), mean fast time (proportion of time that the insects remained in high activity: distance walked > 0.4 cm/frame), and group density network (quantitative measure of social interaction within the group, representing the proximity connections between individuals). Each group of bees from the same pesticide treatment (CTRL, ACE, EOE, GLY, and MET) and social-level tests (colony or individual level) were filmed together, resulting in a total of 30 videos (five pesticide treatments × three replicates × two social levels). Each group of bees was placed in a transparent Petri dish (140 mm diameter × 20 mm height) in a dark room with an artificial light source and filmed for 10 min. The videos were recorded using a webcam (c922 Pro Stream Webcam, Logitech) and Logitech Capture software (version 2.08.11, Logitech) at 30 fps with full HD.

### Data analysis

All statistical analyses were performed in R (version 4.3.2; R Core Team, 2023), and figures were generated using ggplot2 (Wickham 2016). Because the number of replicates per treatment was low (*N* = 3 colonies), we used generalized linear models (GLMs) rather than generalized linear mixed-effects models (GLMMs). GLMs were used as the primary inferential framework to test the effects of pesticide treatments on all continuous and count-based endpoints. All GLMs were implemented using the GAMLSS framework (Rigby and Stasinopoulos 2005) to accommodate non-normal error distributions and ensure appropriate model fit based on diagnostic evaluations. No logarithmic transformation or explicit normalization was applied to the raw data. The error distribution families and link functions were as follows:

(1) Resting time was modeled using an exponential Gaussian (exGAUS) distribution with link functions µ (identity), σ (log), and ν (log);

(2) All other response variables were modeled using a normal (NO) distribution with µ (identity) and σ (log) link functions.

All statistical comparisons were restricted to each pesticide treatment (ACE, EOE, GLY, or MET) versus the control (CTRL), and no pairwise comparisons among pesticide treatments were performed. This approach was adopted because each pesticide was tested at its recommended field-realistic concentration, resulting in substantial and intentional differences in active-ingredient levels among treatments. Under these conditions, direct statistical comparisons among pesticides would confound concentration-dependent effects with compound-specific toxicity and mode of action. Consequently, our analysis was designed to assess whether each pesticide, when applied at its field-recommended concentration, differed from the untreated control. We acknowledge that this approach limits our ability to infer the relative toxicity of the tested pesticides, and that observed differences among treatments may reflect intrinsic sensitivity, mode of action, or exposure concentration. Comparative assessments of pesticide toxicity would require a standardized concentration or dose-response framework, which was beyond the scope of the present study.

Students’ t-tests were used as complementary, within-treatment analyses. Specifically, t-tests were applied to directly compare responses between contaminated and uncontaminated food sources, or between colony- and individual-level responses within the same pesticide treatment, where the comparison involved two clearly defined groups and where model-based comparisons would not provide additional interpretative value. This approach allowed us to maintain a clear separation between model-based inference and targeted pairwise contrasts.

To assess the effects of pesticide exposure on syrup consumption, we fitted GLMs separately for each pesticide treatment (CTRL, ACE, EOE, GLY, or MET) and social level (colony or individual). In these models, the amount of food ingested was used as the response variable, while food type (uncontaminated vs. contaminated honey syrup) was included as a categorical explanatory variable and time (days 1-4) as a numerical explanatory variable. Within each treatment, differences in food ingestion between contaminated and uncontaminated feeders were further evaluated using Student’s t-tests, allowing direct comparison of feeder preference or avoidance within the same exposure context.

At the colony level, we evaluated the effect of pesticide exposure on mortality and colony performance using GLMs. Specifically, we analyzed how the number of dead bees found outside the nest (response variable) was influenced by pesticide treatment (categorical explanatory variable: CTRL, ACE, EOE, GLY, or MET). We also fitted two separate GLMs to assess changes in colony weight gain (response variable): (1) as a function of pesticide treatment, and (2) as a function of the total amount of food ingested (contaminated and uncontaminated syrup combined).

At the individual level, we tested the effect of each pesticide treatment (CTRL, ACE, EOE, GLY, or MET) on bee survival using Kaplan-Meier analysis to estimate survival curves and median survival times (LT_50_). Similarity between the curves was tested using the log-rank test, and comparisons were made using Bonferroni correction (*p* < 0.05). Data were right censored for bees that survived the full period, as they were then used for behavioral analysis and not monitored further. We did not compare the pesticide-contaminated treatments; we only compared the ACE, EOE, GLY, or MET curves with the CTRL curve. We used the R packages “survival” (Therneau and Grambsch 2000; Therneau 2024), “survminer” (Kassambara et al. 2024), and “dplyr” (Wickham et al. 2023).

Behavioral data were obtained using Ethoflow^®^ software, which employs computer vision and artificial intelligence (AI) techniques to track and quantify insect behavior (Bernardes et al. 2021). An AI-based k-means algorithm combined with combinatorial optimization was used to measure behavioral variables while maintaining individual identities within groups (Bernardes et al. 2021). Behavioral variables were analyzed using GLMs to determine how each parameter (“distance walked” [DW], “mean walking speed” [MS], “meandering” [ME], “resting time” [RT], “mean movement time” [MT], “mean fast time” [MF], and “group density network” [GN]) was affected by pesticide treatment (CTRL, ACE, EOE, GLY, or MET) at each social level (colony or individual). Within each treatment, differences between colony- and individual-level behavioral responses were assessed using Student’s t-tests. The EOE treatment was excluded from individual-level analyses of the group density network due to an insufficient number of surviving bees per group (minimum requirement = two individuals).

## Results

### Colony-level food avoidance

**Fig. 1:**
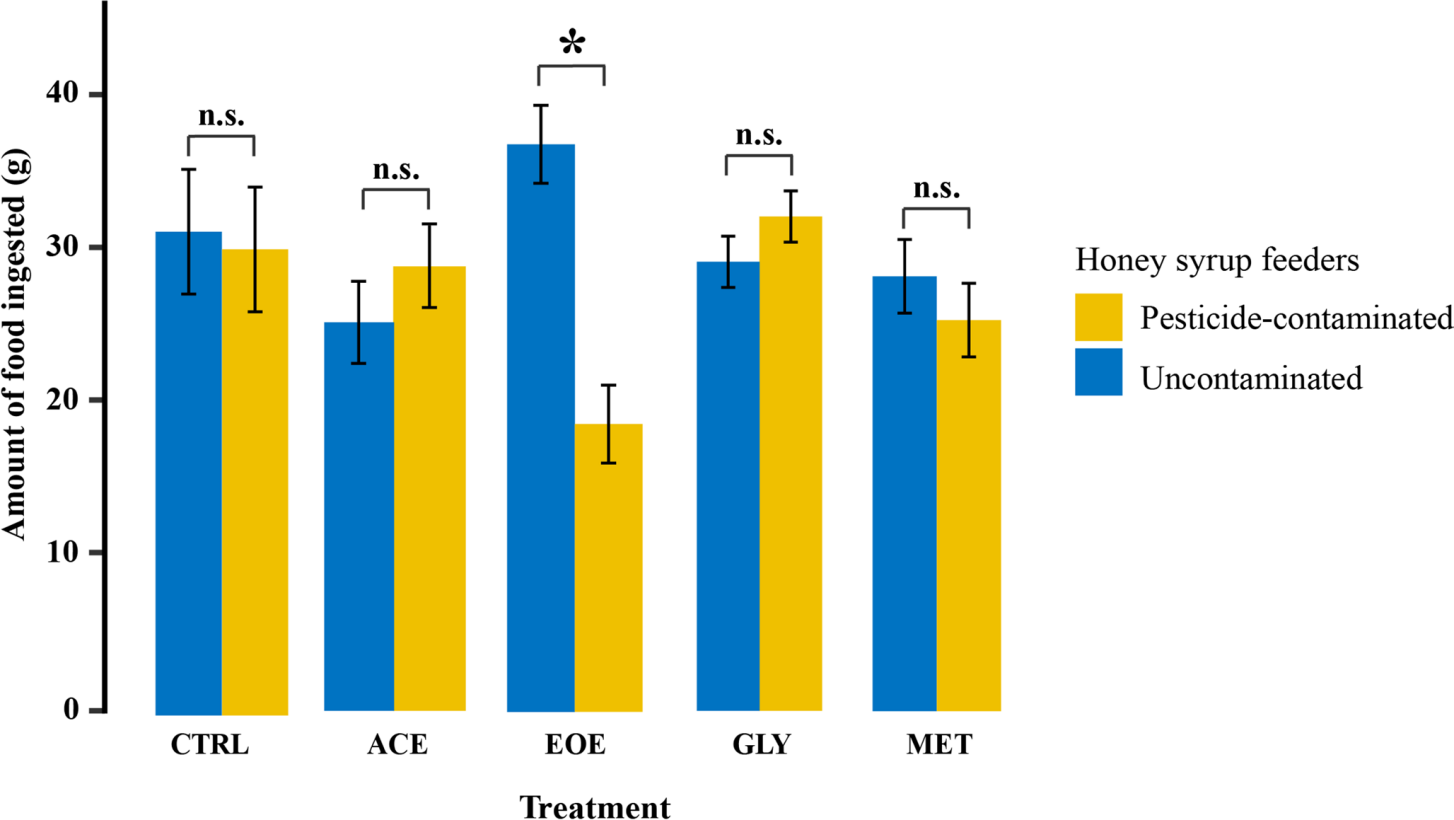
Mean amount of pesticide-contaminated and uncontaminated honey syrup ingested by *Bombus terrestris* colonies after four days of avoidance test. Treatments consisted of one feeder with uncontaminated honey syrup and one feeder with one of the following solutions: (CTRL) uncontaminated honey syrup (control); (ACE) honey syrup contaminated with 0.01 µg/ml of acetamiprid; (EOE) honey syrup contaminated with 476 µg/ml of sweet orange essential oil; (GLY) honey syrup contaminated with 30 µg/ml of glyphosate; or (MET) honey syrup contaminated with 0.05 µg/ml of metalaxyl-M. Each colony was considered a replicate, with three replicates per treatment, resulting in a total of 15 studied colonies. Asterisk indicates significant and “n.s.” indicate not significant differences between the ingestion of pesticide-contaminated and uncontaminated honey syrup within each treatment (*p* < 0.05) group. Error bars represent standard error of the mean (SEM)

At the colony level, no food avoidance was detected in the CTRL, ACE, GLY, or MET treatments, as indicated by the absence of significant differences in consumption between contaminated and uncontaminated honey syrup (CTRL: χ² = 0.042, df = 1, *p* = 0.841; ACE: χ² = 0.934, df = 1, *p* = 0.334; GLY: χ² = 1.557, df = 1, *p* = 0.212; MET: χ² = 0.725, df = 1, *p* = 0.394; Fig. 1). In contrast, colonies exposed to the EOE treatment consumed significantly less contaminated honey syrup than uncontaminated honey syrup (χ² = 18.209, df = 1, *p* < 0.001; Fig. 1). Total food ingestion per day, regardless of contamination status, did not change over time (days 1-4) in any treatment group (CTRL: χ² = 0.141, df = 1, *p* = 0.707; ACE: χ² = 0.666, df = 1, *p* = 0.414; EOE: χ² = 1.287, df = 1, *p* = 0.257; GLY: χ² = 0.616, df = 1, *p* = 0.432; MET: χ² = 1.037, df = 1, *p* = 0.308).

### Individual-level food avoidance

**Fig. 2:**
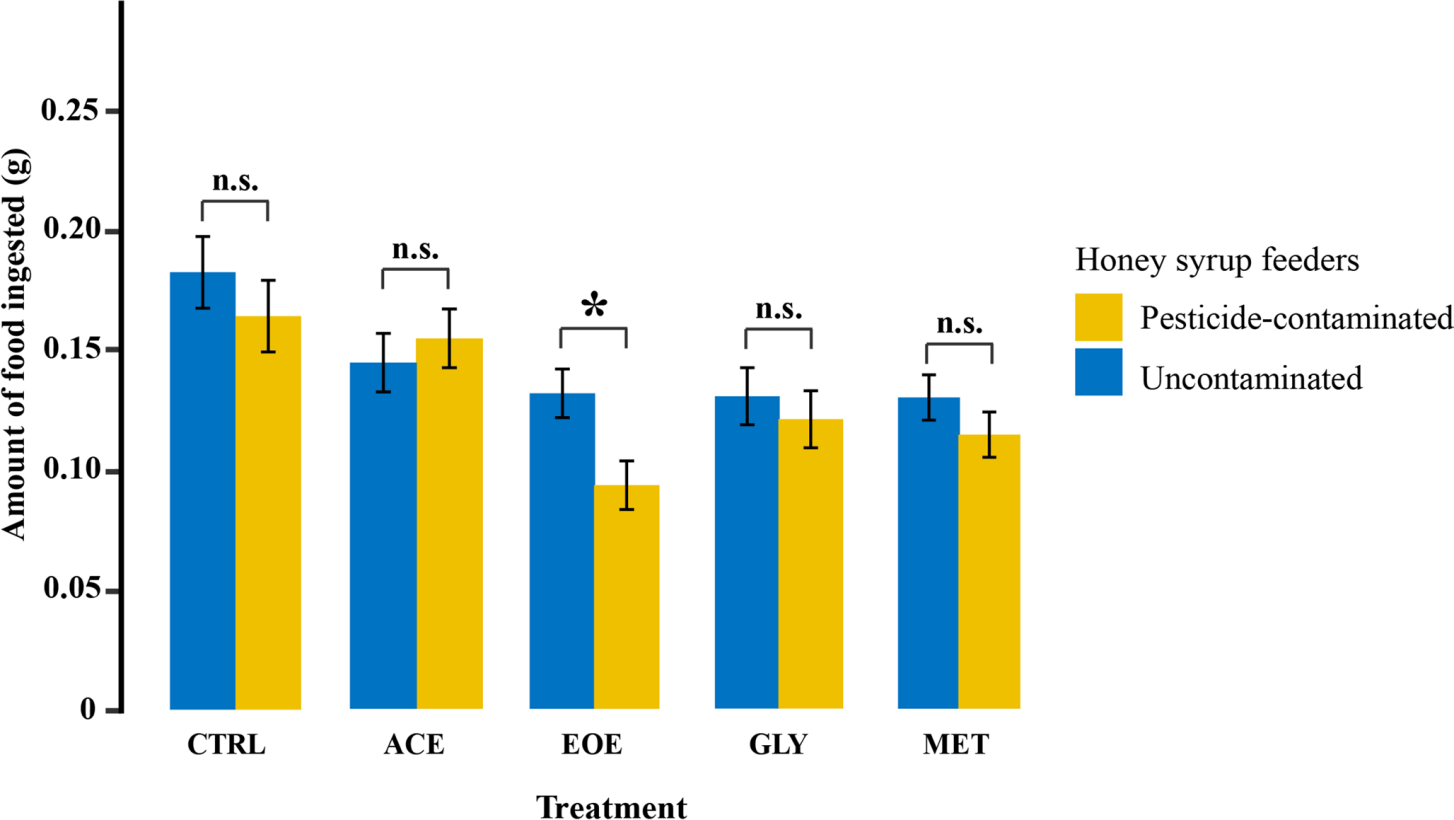
Mean amount of pesticide-contaminated and uncontaminated honey syrup ingested by *Bombus terrestris* individuals after four days of exposure. Treatments consisted of one feeder with uncontaminated honey syrup and one feeder with one of the following solutions: (CTRL) uncontaminated honey syrup (control); (ACE) honey syrup contaminated with 0.01 µg/ml of acetamiprid; (EOE) honey syrup contaminated with 476 µg/ml of sweet orange essential oil; (GLY) honey syrup contaminated with 30 µg/ml of glyphosate; or (MET) honey syrup contaminated with 0.05 µg/ml of metalaxyl-M. Each colony was considered a replicate, with three replicates per treatment, resulting in a total of 15 studied colonies. For each colony, we collected five workers for the individual-level test, and thus, 75 individuals were observed. The asterisk indicates significant and “n.s.” indicate not significant differences between the ingestion of pesticide-contaminated and uncontaminated honey syrup within each treatment (*p* < 0.05) group. Error bars represent standard error of the mean (SEM)

At the individual level, no significant food avoidance was observed for bees exposed to ACE, GLY, or MET when compared with CTRL bees (CTRL: χ² = 0.761, df = 1, *p* = 0.383; ACE: χ² = 0.345, df = 1, *p* = 0.557; GLY: χ² = 0.324, df = 1, *p* = 0.569; MET: χ² = 1.364, df = 1, *p* = 0.243; Fig. 2). Similarly to the colony-level results, bees exposed to EOE consumed significantly less contaminated honey syrup than uncontaminated honey syrup (χ² = 7.024, df = 1, *p* = 0.008; Fig. 2). Total syrup consumption did not vary over time (days 1-4) in any treatment group (CTRL: χ² = 0.141, df = 1, *p* = 0.707; ACE: χ² = 0.666, df = 1, *p* = 0.414; EOE: χ² = 1.287, df = 1, *p* = 0.257; GLY: χ² = 0.616, df = 1, *p* = 0.432; MET: χ² = 1.037, df = 1, *p* = 0.308).

### Lethal and sublethal effects

#### Effects on bee mortality, colony weight, and individual survival

At the colony level, the number of dead bees outside the nest ranged from 0 to 33 and did not differ among treatments (χ² = 1.325, df = 4, *p* = 0.857). Colony weight change over the four-day exposure period ranged from 9.02 g lost to 99.78 g gained and was not affected by pesticide treatment (χ² = 3.245, df = 4, *p* = 0.518) or by the total amount of honey syrup ingested (χ² = 0.129, df = 1, *p* = 0.72).

**Fig. 3:**
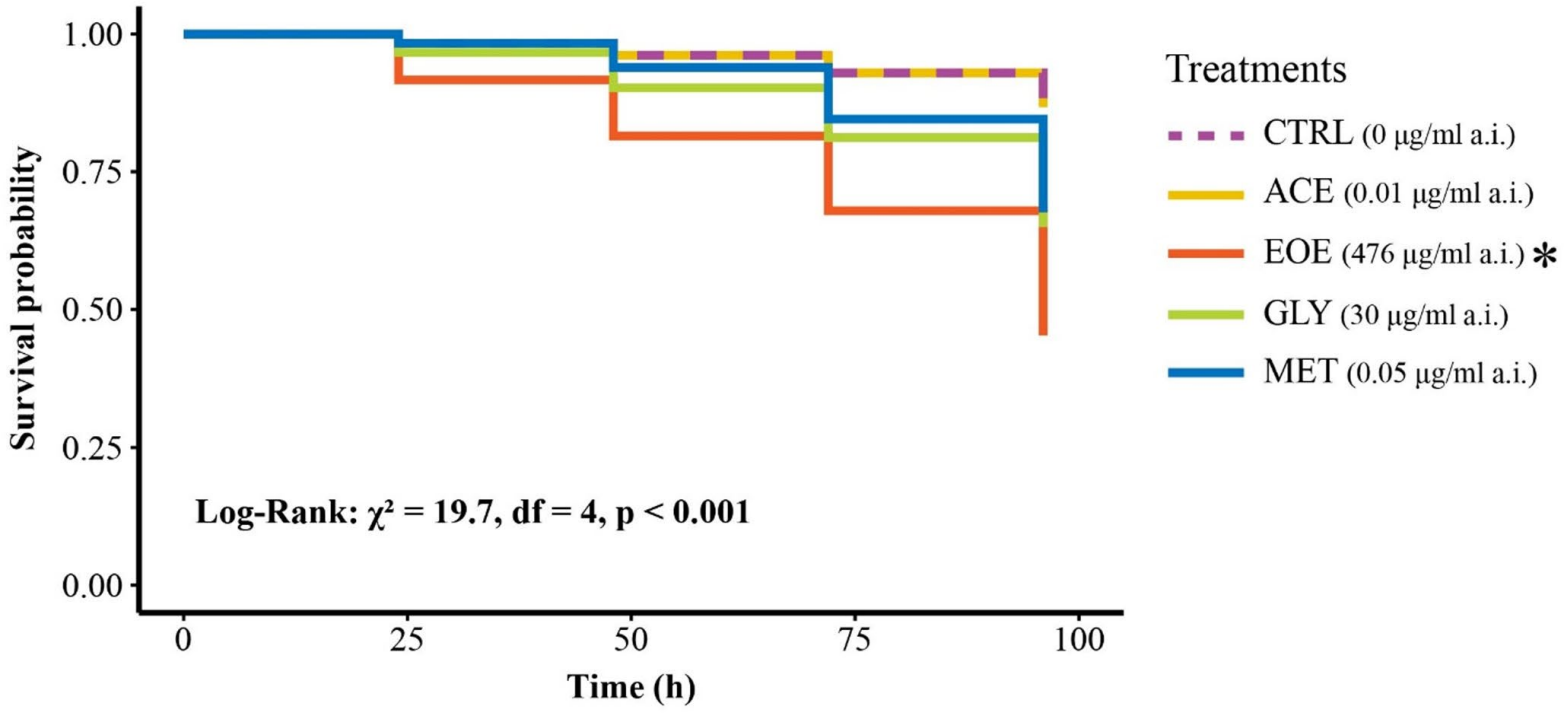
Survival probability of *Bombus terrestris* individuals after four days of avoidance tests with different pesticides (treatments). Treatments consisted of one feeder with uncontaminated honey syrup or one feeder with one of the following solutions: (CTRL) uncontaminated honey syrup (control); (ACE) honey syrup contaminated with 0.01 µg/ml of acetamiprid; (EOE) honey syrup contaminated with 476 µg/ml of sweet orange essential oil; (GLY) honey syrup contaminated with 30 µg/ml of glyphosate; or (MET) honey syrup contaminated with 0.05 µg/ml of metalaxyl-M. Each colony was considered a replicate, with three replicates per treatment, resulting in a total of 15 studied colonies. For each colony we collected five workers for the individual-level test, and thus, a total of 75 individuals were observed. Survival curves were compared using a Log-Rank test (X^2^ = 19.7, df = 4, *p* < 0.001). Asterisk indicates significant difference in survival compared to control (*p* < 0.05)

At the individual level, survival differed among treatments (χ² = 19.7, df = 4, *p* < 0.001; Fig. 3). Survival in the ACE, GLY, and MET treatments did not differ significantly from the CTRL (ACE: χ² = 0, df = 1, *p* = 1; GLY: χ² = 3.5, df = 1, *p* = 0.632; MET: χ² = 2, df = 1, *p* = 1). In contrast, bees exposed to the EOE treatment exhibited significantly reduced survival relative to the CTRL (χ² = 11.7, df = 1, *p* = 0.006; Fig. 3). Median lethal time (LT₅₀) could only be estimated for the EOE treatment (96 h), as survival remained high in the other groups throughout the experimental period.

#### Walking behavior and group density network of bees submitted to the avoidance tests

**Fig. 4:**
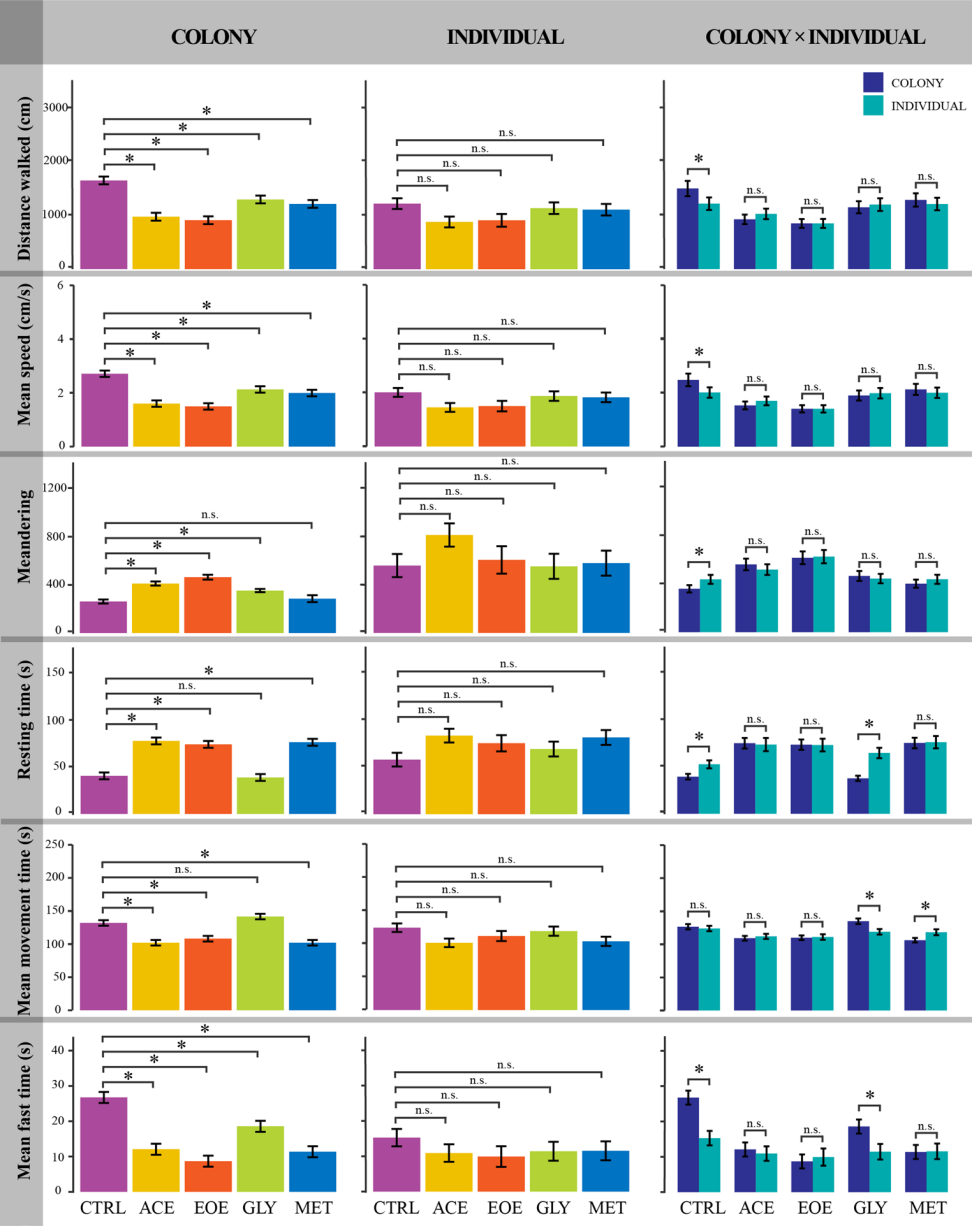
Walking behavior of *Bombus terrestris* workers after four days of avoidance tests with different pesticides (treatments) at colony and individual-level exposure. Treatments consisted of one feeder with uncontaminated honey syrup or one feeder with one of the following solutions: (CTRL) uncontaminated honey syrup (control); (ACE) honey syrup contaminated with 0.01 µg/ml of acetamiprid; (EOE) honey syrup contaminated with 476 µg/ml of sweet orange essential oil; (GLY) honey syrup contaminated with 30 µg/ml of glyphosate; or (MET) honey syrup contaminated with 0.05 µg/ml of metalaxyl-M. Each colony was considered a replicate, with three replicates per treatment, resulting in a total of 15 colonies studied. For each colony we collected five workers for the individual-level test, thus a total of 75 individuals were collected, and the 62 surviving individuals were used for the behavioral bioassay. “Colony” comprises bees tested in a social context; Individual: isolated bees; “Colony × Individuals”: comparisons between both social contexts. For the “Colony” and “Individual” columns, asterisks indicate significant difference between the pesticide-contaminated treatment (ACE, EOE, GLY, or MET) and the CTRL (*p* < 0.05) groups. For the “Colony x Individual” column, asterisks indicate significant and “n.s.” indicate not significant differences within treatments (CTRL, ACE, EOE, GLY, or MET) between bees exposed at colony-level and those exposed at the individual-level (*p* < 0.05). Error bars represent standard error of the mean (SEM)

**Fig. 5:**
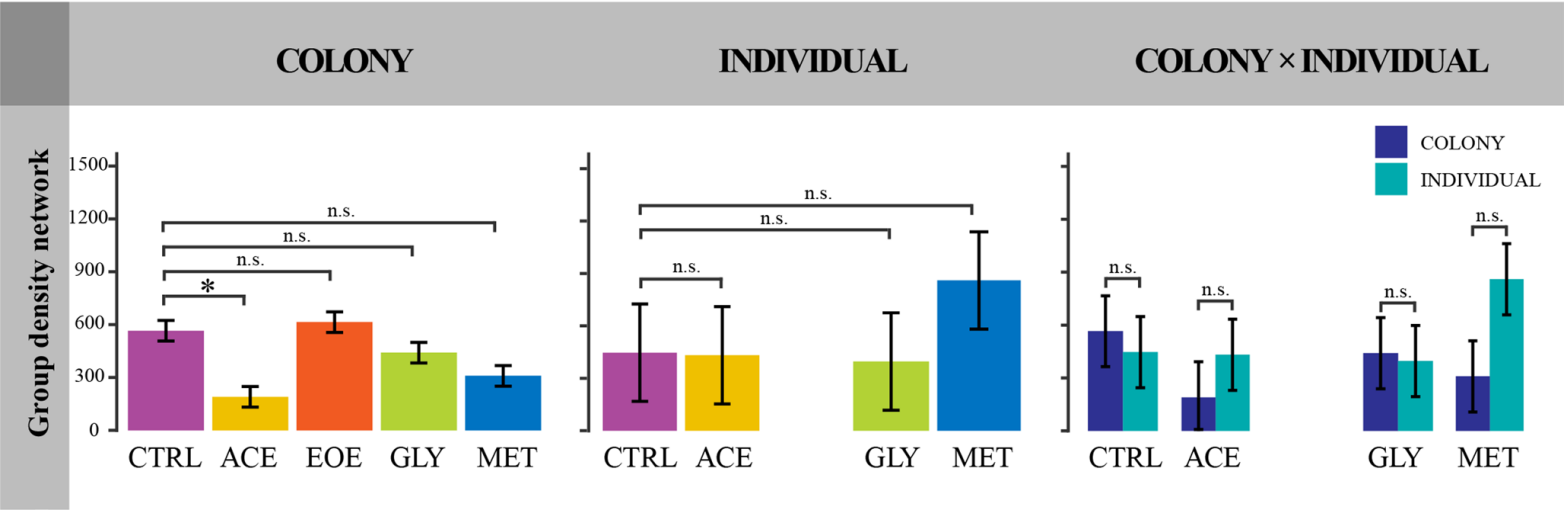
Group density network of *Bombus terrestris* workers after four days of avoidance tests with different pesticides (treatments) at colony and individual-level exposure. Treatments consisted of one feeder with uncontaminated honey syrup or one feeder with one of the following solutions: (CTRL) uncontaminated honey syrup (control); (ACE) honey syrup contaminated with 0.01 µg/ml of acetamiprid; (EOE) honey syrup contaminated with 476 µg/ml of sweet orange essential oil; (GLY) honey syrup contaminated with 30 µg/ml of glyphosate; or (MET) honey syrup contaminated with 0.05 µg/ml of metalaxyl-M. Each colony was considered a replicate, with three replicates per treatment, resulting in a total of 15 colonies studied. For each colony we collected five workers for the individual-level test, thus, a total of 60 individuals were collected, and 52 surviving individuals were used for the behavioral bioassay, not including those from the EOE treatment group. “Colony” comprises bees tested in a social context; Individual: isolated bees; “Colony × Individuals”: comparisons between both social contexts. For the “Colony” and “Individual” columns, asterisks indicate significant and “n.s.” indicate not significant differences between the pesticide-contaminated treatment (ACE, GLY, and MET) and the CTRL (*p* < 0.05) groups. For the “Colony × Individual” column, asterisks indicate significant differences within treatments (CTRL, ACE, GLY, or MET) between bees exposed at colony-level and those exposed at the individual-level (*p* < 0.05). Error bars represent standard error of the mean (SEM)

At the colony level, pesticide treatment significantly affected all measured behavioral parameters, including distance walked, mean walking speed, meandering, resting time, mean movement time, mean fast time, and group density network (Figs. 4 and 5; Supplemental Material S1). Overall patterns indicated reduced locomotor activity and altered movement dynamics in pesticide-exposed colonies relative to the CTRL, although the magnitude and direction of these effects varied among treatments (Supplemental Material S1).

At the individual level, pesticide treatment had no significant effect on any of the behavioral parameters measured (Figs. 4 and 5; Supplemental Material S1). The EOE treatment was excluded from the individual-level group density network analysis due to insufficient numbers of surviving bees.

Comparisons between social levels (colony vs. individual exposure) revealed treatment-specific differences in behavioral responses, most notably in the CTRL group (Figs. 4 and 5; Supplemental Material S1). CTRL bees exposed in the colony walked farther, moved faster, showed less meandering, and had shorter resting time than those individually exposed (Figs. 4 and 5; Supplemental Material S1).

## Discussion

In this study, we provided evidence that individuals and colonies of bumble bees showed no avoidance to pesticide-contaminated food, except for the field concentration of the orange essential oil (476 µg a.i./ml). However, even with this treatment, the bees still ingested pesticide-contaminated food, which led to lower survival and behavioral alterations. We also showed that, despite the lack of lethal effects after the ingestion of the other pesticides, colony exposure to residual concentrations of acetamiprid (0.01 µg a.i./ml), glyphosate (30 µg a.i./ml), and metalaxyl-M (0.05 µg a.i./ml) indicates disruptions to many different behaviors of the bee workers.

Previous studies have demonstrated that bumble bees can be attracted to or be indifferent to some neonicotinoid-contaminated foods (Kessler et al. 2015; Arce et al. 2018; Muth et al. 2020; Catania et al. 2024). Differences among these studies may be due to the species of bumble bees being examined (Muth et al. 2020: *B. impatiens*; Kessler et al. 2015; Arce et al. 2018; Catania et al. 2024; and present study: *B. terrestris*), neonicotinoids and concentrations tested (Muth et al. 2020; imidacloprid; Kessler et al. 2015; imidacloprid, thiamethoxam, and clothianidin; Arce et al. 2018; thiamethoxam; Catania et al. 2024; and present study: acetamiprid). Although *B. terrestris* displays a preference for imidacloprid-contaminated foods (Kessler et al. 2015), this was not observed in *B. impatiens* (Muth et al. 2020). In addition, *B. terrestris* has been shown to prefer foods contaminated with imidacloprid or thiamethoxam, but not with clothianidin (Kessler et al. 2015). The ingestion of higher concentrations of acetamiprid (161.6 µg a.i./ml) elicit anti-feeding behavior on bumble bees (Catania et al. 2024), which was not observed at the concentration tested in the present study (0.01 µg a.i./ml). Our results add to the argument that the foraging behavior of bumble bees towards neonicotinoid-contaminated food is variable, and more studies on this dynamic are necessary to understand the probability of exposure of pollinators to these pesticides in the field.

Bumble bees did not avoid the commercial formulation of sweet orange essential oil here tested, as the bees continued to ingest the biopesticide-contaminated food. In our trials, however, they ate more uncontaminated honey syrup than food treated with the biopesticide, an unexpected result, because plant extracts of *Citrus* tend to be very attractive to bees (Grajales-Conesa et al. 2012; Nurdiansyah et al. 2024). The main compound found in the biopesticide here tested is limonene; however, at least eight other compounds and butylated hydroxytoluene (BHT) are present (Reyes-Ávila et al. 2023). The co-formulants present in this commercial formulation may be responsible for the lower attractiveness of the essential oil. Azadirachtin, another limonoid used as a biopesticide, repels bees and induces anti-feeding behavior (Bernardes et al. 2017). Therefore, the inappropriate use of biopesticides during the plant flowering stage, in addition to posing a risk to bee health (Campolo et al. 2020; Cappa et al. 2022; Catania et al. 2023), can decrease pollination services for sprayed crops, leading to lower fruit and seed production (Catarino et al. 2019).

A lack of avoidance by bumble bees in plants sprayed with glyphosate has already been demonstrated (Thompson et al. 2022; Motta and Moran 2023). However, we showed that they were not attracted to glyphosate-contaminated honey syrup, as found in honey bees (Liao et al. 2017; Almasri et al., 2021a) and stingless bees (Apidae: Meliponini) (Ferreira et al. 2024). As for neonicotinoids, the preference for herbicide-contaminated food might depend on the bee species, the type of herbicide, and the tested concentrations (Almasri et al., 2021b). As this is the first assessment of bumble bee preference for herbicide, further studies should be conducted to investigate this hypothesis.

This study is also the first to assess the avoidance of fungicides in bumble bees and the first to use metalaxyl-M for all bee species. We showed that *B. terrestris* did not avoid foods contaminated with metalaxyl-M when given a choice of uncontaminated food. For neonicotinoids and herbicides, the preferred response of bees seemed to depend on the bee species and active ingredients tested. Honey bees tend to avoid fungicide-contaminated food (Kang and Jung 2017), although a preference for chlorothalonil-contaminated food has also been observed (Liao et al. 2017).

Despite being given a choice of uncontaminated food, bees from all pesticide-contaminated treatment groups consumed contaminated honey syrup to some degree, which caused significant lethal or sublethal effects. The detrimental effects of pesticides on bumble bees subjected to preference tests have also been reported by Kessler et al. (2015) and Muth et al. (2020). These observations show that bumble bees, even without a preference for pesticide-contaminated food, ingest sufficient quantities of pesticides that can cause individual- and colony-level effects with potential implications for colony failure, bee population decline, and reduced pollination services (Stanley et al. 2015; Crall et al. 2019; Demirozer et al. 2022; Nicholson et al. 2024).

Moreover, bumble bees responded differently to the treatments when subjected to tests at the colony and individual levels. We detected lethal effects only when bees were individually treated with pesticides. In contrast, sublethal effects occurred only in experiments performed at the colony level. Thus, the lethal effects of the biopesticides tested on individuals could be buffered by the colony, maybe due to social behaviors such as social immunity (Gill et al. 2012; Becher et al. 2018; Crall et al. 2019). These findings emphasize that standard laboratory protocols using isolated individuals may underestimate the real-world impacts of pesticides on eusocial insects. In addition, all pesticides altered the walking behavior of bees exposed at the colony level, indicating a tendency toward increased resting and reduced speed, movement, and distance walked. Non-treated bees (CTRL) also displayed reduced locomotion when isolated compared to control bees kept in their colonies. Because CTRL bees were only exposed to uncontaminated honey syrup, we assumed that the detrimental effects observed were due to isolation from the colony over four days. Social insects subjected to social isolation may display behavioral, morphological, and physiological alterations (Breed, 1983; Scharf et al. 2021; Wang et al. 2022). The different results found for tests performed at the colony or individual levels, in addition to the lack of behavioral differences between treated and non-treated individual bees, suggest that social isolation can mask the effect of pesticide exposure in bumble bees. However, this hypothesis must be further tested with experiments taking into consideration bees’ isolation independently of pesticide exposure.

Generally, combinations of stressors on bees tend to be considered additive or synergistic (Henry et al. 2017; Botías et al. 2021). Some stressors, however, can have antagonistic or non-additive interactions effects on bees, where one stressor may mask or reduce the physiological response of bees in relation to a particular compound (Dickel et al. 2018; Straub et al. 2022). Our results suggested a non-additive effect of social isolation and pesticide exposure on individual bumble bees subjected to the avoidance test. This highlights the importance of considering the social context when performing pesticide risk assessments for eusocial insects. If we had evaluated the behavioral differences between the CTRL and pesticide-contaminated treatment groups only for bees exposed at the individual level, we would not be able to find the alterations caused by the pesticides in walking behavior and group density network, which were only observed in workers exposed in the colony.

Acetamiprid was the only pesticide that decreased social interactions, which has been previously observed in neonicotinoids (Boff et al. 2018). All these sublethal effects caused by neonicotinoid, biopesticide, herbicide and fungicide here tested reinforces the arguments raising doubts on the safety of non-insecticide and natural agrochemicals to pollinators (Barascou et al. 2021; Battisti et al. 2021; Cappa et al. 2022; Catania et al. 2023). Thus, our results suggest that more holistic protocols are necessary to perform adequate risk assessments for social pollinators.

## Conclusions

Bumble bees are at high probability of pesticide exposure in the field because they are unable to avoid food contaminated with diverse groups of pesticides, including neonicotinoids, biopesticides, herbicides, and fungicides. Even when given a choice of uncontaminated food, they forage for food contaminated with pesticides, which can decrease their survival and have detrimental effects on their locomotion and group network. Therefore, future risk assessments protocols must integrate social context and multiple pesticide classes to better reflect real-world exposure and risk to pollinators.

## Supplementary Information

Below is the link to the electronic supplementary material.

Supplementary Material 1

## Data Availability

The datasets generated during and/or analyzed during the current study are available from the corresponding author on reasonable request.
