# Supplementary material for: Absence of pesticide avoidance during chronic colony-level exposure modifies locomotor activity in bumble bees
Source: Ecotoxicology. 2026 Feb 21;35(3):65. doi: 10.1007/s10646-026-03045-4 (PMC12924873; doi:10.1007/s10646-026-03045-4)
Supplement: Supplementary file 1 — Supplementary Material 1 [file 10646_2026_3045_MOESM1_ESM.pdf]

Table S1. Statistical test results for behavioral parameters of bumblebees in the avoidance tests. The table shows the test type (χ^2 or t), test statistic (value), degrees of freedom (df), and p-values for each parameter ("distance walked" [DW], "mean walking speed" [MS], "meandering" [ME], "resting time" [RT], "mean movement time" [MT], "mean fast time" [MF], and "group density network" [GN]) at the colony level, individual level, and for comparisons between colony- and individual-level tests of each parameter per treatment (CTRL – uncontaminated honey syrup [control]; ACE – 0.01 µg/ml of acetamiprid; EOE – 476 µg/ml of sweet orange essential oil; GLY – 30 µg/ml of glyphosate; or MET – 0.05 µg/ml of metalaxyl-M).

Level/Comparison	Parameter	Test	Value	df	p-value
Colony-level	DW	χ^2	47.082	4	<0.001*
Colony-level	MS	χ^2	47.083	4	<0.001*
Colony-level	ME	χ^2	50.599	4	<0.001*
Colony-level	RT	χ^2	71.698	4	<0.001*
Colony-level	MT	χ^2	52.974	4	<0.001*
Colony-level	MF	χ^2	57.564	4	<0.001*
Colony-level	GN	χ^2	18.504	4	<0.001*
Individual-level	DW	χ^2	7.625	4	0.106
Individual-level	MS	χ^2	7.6	4	0.107
Individual-level	ME	χ^2	4.79	4	0.309
Individual-level	RT	χ^2	7.304	4	0.121
Individual-level	MT	χ^2	8.266	4	0.082
Individual-level	MF	χ^2	2.477	4	0.648
Individual-level	GN	χ^2	1.721	3	0.632
Colony vs Individual – CTRL	DW	t	2.524	125	0.013*
Colony vs Individual – CTRL	MS	t	2.529	125	0.013*
Colony vs Individual – CTRL	ME	t	-2.494	125	0.014*
Colony vs Individual – CTRL	RT	t	-2.536	125	0.012*

Colony vs Individual – CTRL	MT	t	0.54	125	0.59
Colony vs Individual – CTRL	MF	t	4.024	125	<0.001*
Colony vs Individual – CTRL	GN	t	0.417	125	0.682
Colony vs Individual – ACE	DW	t	-1.243	125	0.216
Colony vs Individual – ACE	MS	t	-1.245	125	0.215
Colony vs Individual – ACE	ME	t	0.987	125	0.326
Colony vs Individual – ACE	RT	t	0.163	125	0.871
Colony vs Individual – ACE	MT	t	-0.61	125	0.543
Colony vs Individual – ACE	MF	t	0.409	125	0.684
Colony vs Individual – ACE	GN	t	-0.848	125	0.41
Colony vs Individual – EOE	DW	t	0.002	125	0.999
Colony vs Individual – EOE	MS	t	0.0	125	1.000
Colony vs Individual – EOE	ME	t	-0.185	125	0.853
Colony vs Individual – EOE	RT	t	0.041	125	0.968
Colony vs Individual – EOE	MT	t	-0.226	125	0.821
Colony vs Individual – EOE	MF	t	-0.393	125	0.695
Colony vs Individual – GLY	DW	t	-0.501	125	0.617
Colony vs Individual – GLY	MS	t	-0.503	125	0.616
Colony vs Individual – GLY	ME	t	0.572	125	0.568
Colony vs Individual – GLY	RT	t	-4.348	125	<0.001*
Colony vs Individual – GLY	MT	t	3.041	125	0.003*
Colony vs Individual – GLY	MF	t	2.402	125	0.012*
Colony vs Individual – GLY	GN	t	0.157	125	0.877
Colony vs Individual – MET	DW	t	0.696	125	0.487
Colony vs Individual – MET	MS	t	0.697	125	0.487
Colony vs Individual – MET	ME	t	-1.031	125	0.304
Colony vs Individual – MET	RT	t	-0.1	125	0.921
Colony vs Individual – MET	MT	t	-2.328	125	0.021*
Colony vs Individual – MET	MF	t	-0.062	125	0.951
Colony vs Individual – MET	GN	t	-1.93	125	0.073

* Statistically significant p-value ($p < 0.05$)
